# B-lines by lung ultrasound as a predictor of re-intubation in mechanically ventilated patients with heart failure

**DOI:** 10.3389/fcvm.2024.1351431

**Published:** 2024-02-08

**Authors:** Junho Hyun, Ah-ram Kim, Sang Eun Lee, Min-Seok Kim

**Affiliations:** Division of Cardiology, Department of Internal Medicine, Asan Medical Center, University of Ulsan College of Medicine, Seoul, Republic of Korea

**Keywords:** heart failure, mechanical ventilation, ventilator weaning, ultrasound, pulmonary edema

## Abstract

**Introduction:**

There have been few studies on predictors of weaning failure from MV in patients with heart failure (HF). We sought to investigate the predictive value of B-lines measured by lung ultrasound (LUS) on the risk of weaning failure from mechanical ventilation (MV) and in-hospital outcomes.

**Methods:**

This was a single-center, prospective observational study that included HF patients who were on invasive MV. LUS was performed immediate before ventilator weaning. A positive LUS exam was defined as the observation of two or more regions that had three or more count of B-lines located bilaterally on the thorax. The primary outcome was early MV weaning failure, defined as re-intubation within 72 h.

**Results:**

A total of 146 consecutive patients (mean age 70 years; 65.8% male) were enrolled. The total count of B-lines was a median of 10 and correlated with NT-pro-BNP level (*r*^2 ^= 0.132, *p* < 0.001). Early weaning failure was significantly higher in the positive LUS group (9 out of 64, 14.1%) than the negative LUS group (2 out of 82, 2.4%) (*p* = 0.011). The rate of total re-intubation during the hospital stay (*p* = 0.004), duration of intensive care unit stay (*p* = 0.004), and hospital stay (*p* = 0.010) were greater in the positive LUS group. The negative predictive value (NPV) of positive LUS was 97.6% for the primary outcome.

**Conclusion:**

B-lines measured by LUS can predict the risk of weaning failure. Considering the high NPV of positive LUS, it may help guide the decision of weaning in patients on invasive MV due to acute decompensated HF.

## Introduction

A subset of patients with heart failure (HF) experience episodes of decompensation during their clinical course (1). Approximately 5% of hospitalized patients and 23% of those admitted to the intensive care unit (ICU) due to HF aggravation require invasive mechanical ventilation (MV) because of respiratory failure (2). However, weaning from MV among HF patients is challenging, and a significant proportion of them experience weaning failure or re-intubation (3, 4). During the weaning process, preload and afterload of both ventricles and the work of breathing are abruptly altered, which can increase the risk of early re-intubation in HF patients with poor myocardial reserve (5–7). In order to avoid the need for re-intubation due to failed extubation, it is crucial to optimize the preload by removing excess fluid. An accurate evaluation of any remaining pulmonary congestion is essential for this purpose, which is typically done through the use of chest radiography and natriuretic peptides (NPs). However, over 10% of HF patients on MV still experience re-intubation with these conventional methods (3, 4).

B-lines, which can be easily measured using lung ultrasound (LUS), can detect pulmonary interstitial edema and are correlated with NPs and pulmonary capillary wedge pressure (PCWP), making them useful for diagnosing and predicting outcomes among patients with HF (8–10). However, the clinical value of B-lines in patients receiving invasive MV due to decompensated HF is unclear. While previous studies reported that LUS parameters can predict post-extubation outcomes, most of them focused on non-cardiac populations (11). As B-lines can be used to monitor the adequacy of decongestive therapy (12), they may also provide clinical information about optimizing the preload during ventilatory support in patients with HF. Therefore, we aimed to evaluate the prognostic value of B-lines for in-hospital outcomes in HF patients receiving MV.

## Materials and methods

### Study design and participants

This was a prospective observational cohort study conducted at a tertiary center (Asan Medical Center, Seoul, Korea) between March 2020 and December 2022. This study was registered at ClinicalTrial.gov (identifier NCT04322851) and conducted in compliance with the Declaration of Helsinki. The study protocol was approved by the Institutional Review Board of the center (Asan Medical Center, AMC-2020-0164, approved February 06, 2020), and all of the participating patients provided written informed consent. When patients were unconscious or delirious, their legal representatives provided consent for their participation.

This study included consecutive patients who had received invasive MV for acute decompensated HF despite refractory respiratory failure with supplementary oxygen therapy and non-invasive positive pressure ventilation and were subsequently eligible for MV weaning with planned extubation. Acute decompensated HF was defined as a sudden or gradual worsening of the signs and symptoms of HF, leading to unplanned hospitalization or requiring intravenous therapy to relieve signs and/or symptoms. Patients were included if they met the following MV weaning criteria: (1) fraction of inspired oxygen (FiO_2_) less than 0.4; (2) stable hemodynamic status with no need for or a low level of inotropes and/or vasopressors; and (3) a stable ventilatory state with a spontaneous breathing trial including a respiration rate less than 35, heart rate lower than 140 beats per minute, and an increase of the heart rate by less than 20% of baseline, oxygen saturation higher than 90%, and no signs of respiratory distress (13). Patients were scheduled for extubation upon meeting the specified weaning criteria mentioned above, provided they were cooperative, responsive, and exhibited an adequate coughing reflex. Patients with a tracheostomy state, who received MV not attributable to HF, or who received MV during elective cardiac surgery without evidence of HF were excluded. Most of the spontaneous breathing trials were conducted for approximately one hour using a T-piece. Subsequently, all participants underwent extubation. Baseline demographics, clinical data during the hospitalization, and laboratory findings, including N-terminal pro-brain natriuretic peptide (NT-pro-BNP) on the day of MV weaning, were collected. Arterial blood gas analysis was performed before and after weaning from MV.

### Lung ultrasound

LUS was performed using the Philips CX50 ultrasound system (Philips Medical Systems, Bothell, WA, USA) with a 3.5 MHz convex probe at a depth of 15–18 cm adjusted to the patient's chest wall thickness. The LUS was performed by an investigator (J.H.) who was trained in a standard protocol recommended by the international guideline (14). All patients were examined with LUS immediately before extubation in a semi-recumbent position and without the support of any positive pressure on a T-piece. B-lines were measured by scanning eight regions of the thorax in a longitudinal plane between two ribs with a distance of adjacent two B-lines <7 mm (14, 15). B-lines were defined as linear, vertical hyperechoic artifacts that start from the pleura moving synchronously with respiration. Each hemithorax was divided into upper and lower regions, which were further divided into medial and lateral regions by the anterior axillary line. The scanned images were independently adjudicated by two investigators (J.H. and A.K.) who were blinded to the clinical information. A positive region was defined as the presence of three or more B-line count in each area scanned. A positive LUS examination was defined as having two or more positive regions in both hemithoraces.

### Outcomes

The patients were divided into two groups based on positive for B-lines or not. The primary outcome of the study was the rate of re-intubation within 72 h. Secondary outcomes included the rates of total re-intubation during entire hospital stay, in-hospital death, and the durations of ICU and hospital stays. Re-intubation was performed in those who showed signs of respiratory failure despite adjunctive supplemental oxygen therapy after the extubation process. All clinical and outcome data were collected during the index admission. Outcomes were analyzed according to the total count of B-lines to determine the relationship between B-lines and re-intubation and their correlations with the NT-pro-BNP level.

### Statistical analyses

The rates of re-intubation and in-hospital death were compared using Pearson's Chi-square test or Fisher's exact test, as appropriate. The odds ratio of outcomes were analyzed with logistic regression adjusted with variables including age, hypertension, diabetes, chronic kidney disease, body weight change between intubation and extubation, and left ventricular ejection fraction (LVEF). Inter-observer agreements on positive exams and the total count of B-lines were analyzed by Cohen's kappa measure of concordance and the intraclass correlation coefficient for agreement, respectively. The correlation between the total count of B-lines and NT-pro-BNP with the log-transformed value, considering its skewed distribution, was estimated with the coefficient of determination (*r*^2^) using linear regression. Receiver-operating characteristics (ROC) analysis was used to determine the performance of the B-line result, and the areas under the ROC curve (AUC) are presented with 95% confidence intervals (CI). Sensitivity, specificity, and positive and negative predictive values (PPV and NPV) of the positive LUS were also analyzed. All statistical analyses were performed using IBM SPSS Statistics for Windows, version 22.0 (IBM Corp., Armonk, NY, USA). All comparisons were two-sided, and *P*-values <0.05 were considered statistically significant.

## Results

### Baseline characteristics

A total of 146 consecutive patients who received invasive MV due to congestive HF were enrolled. Sixty-four patients (43.8%) had a positive LUS result (positive LUS group), and the remaining 82 (56.2%) had a negative LUS exam (negative LUS group). In terms of baseline characteristics, the positive LUS group had an older age, lower body weight at the initiation of MV, lower hemoglobin level, and higher NT-pro-BNP level compared with the negative LUS group (Table 1). The proportion of patients with an ischemic etiology of HF was 49.3% (*n* = 72) in the entire cohort, which was not significantly different between the two groups (*p* = 0.851). The study population received invasive MV for a median of 6 days [interquartile range (IQR), 3–12], and the positive LUS group had a longer median duration (8 days) than the negative LUS group (5 days) (*p* = 0.027). Echocardiography was performed at a median of 4 days (IQR, 2–8) before MV weaning, and the measures were similar between the two groups.

**Table 1:** Baseline characteristics of the study population.

	Positive LUS *N* = 64	Negative LUS *N* = 82	*p*-value
Demographic data			
Age, years	72.8 ± 10.0	67.8 ± 13.0	0.012
Male sex	40 (62.5)	56 (68.3)	0.464
Height, cm	163.0 ± 0.1	163.6 ± 0.1	0.714
Body weight, kg^a^	62.8 ± 13.2	67.6 ± 13.2	0.037
Hypertension	39 (60.9)	54 (65.9)	0.540
Diabetes	27 (42.2)	34 (41.5)	0.930
Chronic kidney disease	25 (39.1)	29 (35.4)	0.646
Renal replacement therapy	5 (7.8)	10 (12.2)	0.387
Chronic lung disease	9 (14.1)	9 (11.0)	0.573
Peripheral arterial disease	5 (7.8)	13 (15.9)	0.143
Stroke	8 (12.5)	7 (8.5)	0.434
Prior cardiac surgery	24 (37.5)	23 (28.0)	0.225
Coronary artery disease	25 (39.1)	44 (53.7)	0.080
PCI	13 (20.3)	31 (37.8)	0.022
CABG	7 (11.3)	13 (15.9)	0.433
Causes of HF			
Ischemic	31 (48.4)	41 (50.0)	0.851
ICMP	11 (17.2)	15 (18.3)	0.862
MI	20 (31.3)	26 (31.7)	0.953
Valvular	17 (26.6)	15 (18.3)	0.231
Dilated cardiomyopathy	4 (6.3)	5 (6.1)	1.000
Hypertrophic cardiomyopathy	3 (4.7)	2 (2.4)	0.654
Arrhythmia	3 (4.7)	7 (8.5)	0.513
Others	6 (9.4)	12 (14.6)	0.338
Echocardiographic measures			
Interval, days^b^	6 (2–8)	4 (1–9)	0.744
LVEF, %	40 (28–56)	45 (32–57)	0.368
LVEDD, mm	52 (46–58)	50 (44–56)	0.341
E/A	1.65 ± 1.19	1.31 ± 0.98	0.132
E/e’	19.1 ± 12.9	16.3 ± 9.2	0.156
Septal e’, cm/s	5.4 ± 2.3	5.3 ± 1.9	0.697
Peak TR velocity, m/s	2.5 (2.3–3.1)	2.5 (2.2–2.9)	0.502
Characteristics at extubation			
Duration on ventilator, days	8 (4–13)	5 (3–10)	0.027
PaO_2_/FiO_2_ ratio after extubation, mmHg	265 (210–349)	265 (227–327)	0.814
Body weight, kg	59.4 ± 12.6	64.0 ± 12.2	0.028
Change in body weight^c^	−2.8 (−1.0 to −5.4)	−3.1 (−1.2 to −5.1)	0.451
Laboratory data			
Hemoglobin, mg/dl	9.0 ± 1.4	9.7 ± 1.8	0.008
eGFR, ml/min/1.73 m^2^	55.0 ± 29.8	58.2 ± 35.4	0.547
Sodium mmol/L	138.6 ± 4.9	138.6 ± 4.0	0.938
NT-pro-BNP, ng/L^d^	12,539 (4,795–25,109)	4,916 (2,241–10,549)	0.001
Troponin I, pg/ml^d^	683.5 (90.3–3,799.0)	3,730 (113.8–2,235.0)	0.542
Rhythm			0.176
Sinus rhythm	35 (54.7)	57 (69.5)	
AF or AFL	22 (34.4)	18 (22.0)	
Pacing	6 (9.4)	4 (4.9)	
Others	1 (1.6)	3 (3.7)	

Values are presented as patient number (percentage) or median (IQR) or mean ± standard deviation.

AF, atrial fibrillation; AFL, atrial flutter; CABG, coronary artery bypass graft; e’, early diastolic mitral annular velocity; E/A, early to atrial filling velocity; E/e’, ratio of the early transmitral flow velocity to early diastolic mitral annular velocity; eGFR, estimated glomerular filtration rate; FiO_2_, fraction of inspired oxygen; HF, heart failure; ICMP, ischemic cardiomyopathy; LUS, lung ultrasound; LVEDD, left ventricular end-diastolic dimension; LVEF, left ventricular ejection fraction; MI, myocardial infarction; NT-pro-BNP, N-terminal pro-B-type natriuretic peptide; PaO_2_, partial pressure of oxygen in arterial blood; PCI, percutaneous coronary intervention; TR, tricuspid regurgitation.

^a^ Body weight on the day of initiation of mechanical ventilation.

^b^ Interval between echocardiography and extubation.

^c^ Change in body weight was calculated as body weight at extubation—body weight at intubation.

^d^ Levels of NT-pro-BNP and troponin I were available in 112 and 132 patients, respectively.

### Outcomes

The median total count of B-lines detected by LUS was 10 (IQR, 6–16) (Supplementary Figure S1), and the inter-observer agreement was acceptable [Cohen's kappa coefficient 0.84, confidential interval (CI) 0.74–0.93; intraclass correlation coefficient 0.91, 95% CI 0.83–0.95]. A total B-line count and the proportion of positive regions (≥3 B-lines in the region) were highest in the lower lateral region of the thorax (Supplementary Table S1). The details of the B-lines in each group are provided in Supplementary Table S2. The rate of early re-intubation was significantly higher in the positive LUS group (14.1%) compared with the negative LUS group (2.4%) (*p* = 0.011, Table 2). The rate of re-intubation during the whole period of the index hospitalization was also significantly higher in the positive LUS group (26.6% vs. 8.5%, *p* = 0.004). The durations of hospital stay, total ICU stay, and ICU stay after MV weaning were all longer in the positive LUS group. In-hospital mortality was not significantly different between the two groups.

**Table 2:** Study endpoints according to the B-line result.

Endpoint	Positive LUS *N* = 64	Negative LUS *N* = 82	*p*-value
Re-intubation			
Within 72 h (primary endpoint)	9 (14.1)	2 (2.4)	0.011
During the hospital stay	17 (26.6)	7 (8.5)	0.004
In-hospital mortality	4 (6.3)	3 (3.7)	0.699
Duration of stay, days			
Entire hospital stay	34 (19–56)	24 (12–41)	0.010
ICU	12 (7–21)	9 (5–14)	0.004
ICU stay after extubation	3 (2–6)	2 (1–3)	0.001

Values are presented as patient number (percentage) or median (IQR).

ICU, intensive care unit; LUS, lung ultrasound.

### Association of the count of B-lines with outcomes and NT-pro-BNP level

**Figure 1:**
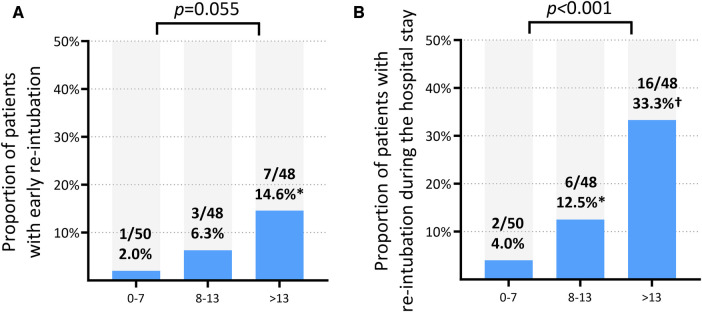
Rates of re-intubation according to the tertiles of total B-line count. (**A**) Re-intubation within 72 h (NP level cannot reliably predict). (**B**) Re-intubation during the entire hospital stay. *p < 0.05 for the comparison with the first tertile (B-line count ≤7). ^†^*p* < 0.001 for the comparison with the first tertile (B-line count ≤7).

When divided into three groups according to the tertile of B-line count, the total count of measured B-lines showed a proportional increase in the rate of the primary outcome, albeit without statistical significance (*p* = 0.055) (Supplementary Table S3). The rate of total re-intubation was significantly different according to the groups divided by the B-line count tertile (*p* < 0.001). Patients in the first tertile (total count of B-lines ≤7) had the lowest risk of re-intubation within 72 h (2.0%) and during the hospital stay (4.0%), while those in the third tertile (total count of B-lines ≥14) had the highest rate of re-intubation within 72 h (14.6%) and during the hospital stay (33.3%) (Figure 1). The NT-pro-BNP level measured on the day of weaning was weakly correlated with a total B-line count (*r*^2 ^= 0.132, *p* < 0.001) in the whole study population and in patients not dependent on dialysis [*n* = 131 (89.7%); *r*^2 ^= 0.170, *p* < 0.001] (Supplementary Figure S2).

### Prediction of re-intubation

**Figure 2:**
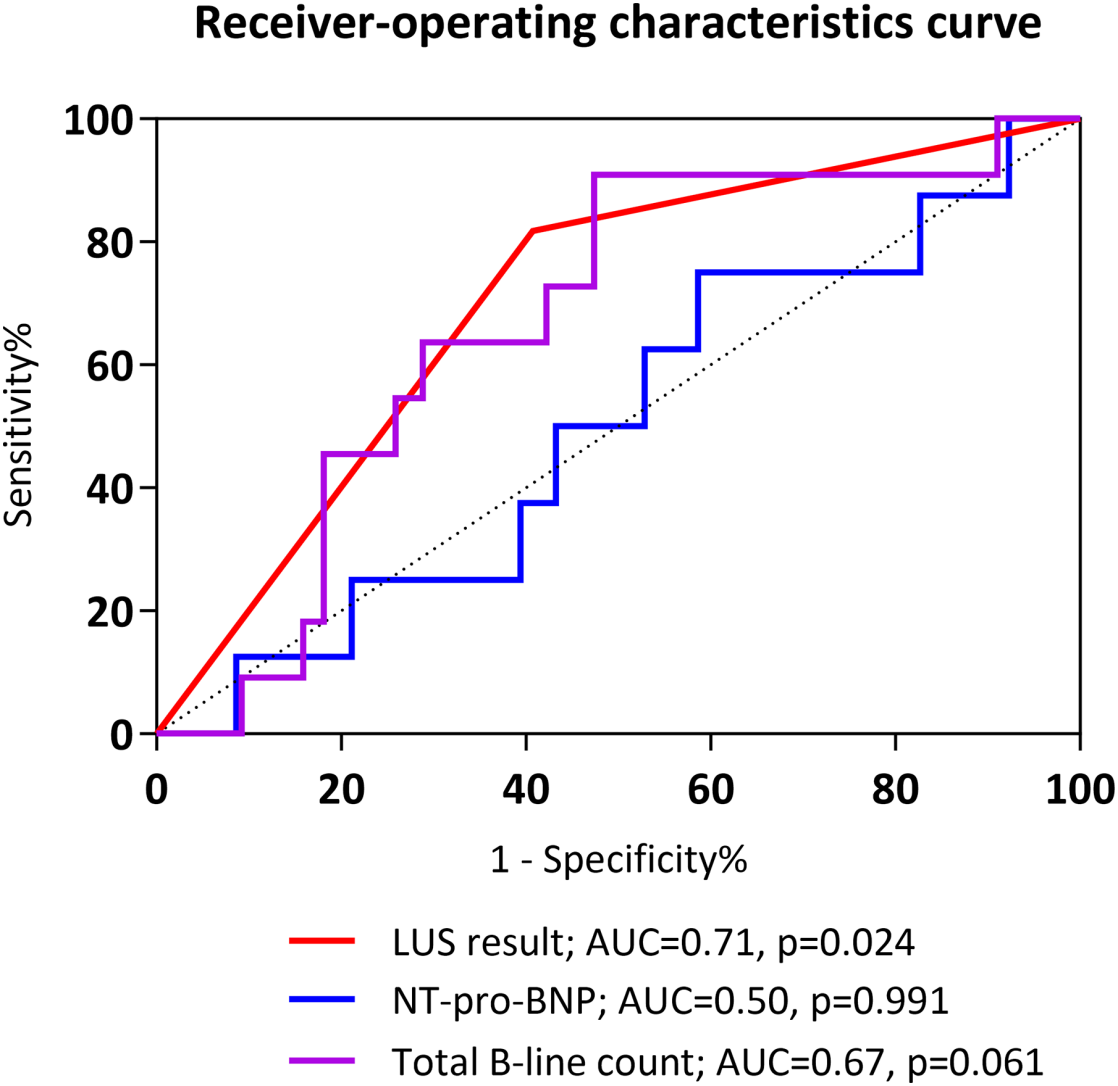
Receiver-operating characteristic curves for prediction of early re-intubation. AUC, area under the receiver operating characteristic curve; NT-pro-BNP, N-terminal pro-B-type natriuretic peptide.

Regression analysis with multivariable adjustment demonstrated that a positive LUS result was associated with re-intubation within 72 h [odds ratio (OR), 6.00; 95% CI, 1.15–31.42; *p* = 0.034] and during the entire hospital stay (OR, 5.54; 95% CI, 1.60–19.18; *p* = 0.007). ROC analysis showed that the AUC value of the B-line result was 0.71 (95% CI, 0.56–0.85; *p* = 0.024) and that of the NT-pro-BNP value was 0.50 (95% CI, 0.31–0.70; *p* = 0.991) (Figure 2). The predictive ability for early re-intubation was better for a positive LUS result than for a total B-line count taken as a continuous variable (Table 3). The NPV of a positive LUS result was 97.6% for early re-intubation and 91.5% for re-intubation during the entire hospital stay (Table 4).

**Table 3:** Predictive value of B-line result for re-intubation using regression analyses.

	Univariable		Multivariable	
	OR (95% CI)	*p*-value	OR (95% CI)	*p*-value
Re-intubation within 72 h				
Positive B-line	6.55 (1.36–31.47)	0.019	6.00 (1.15–31.42)	0.034
Total number of B-line	1.03 (0.97–1.09)	0.343	1.07 (0.96–1.20)	0.243
Age	1.05 (0.98–1.12)	0.149		
Hypertension	0.66 (0.19–2.28)	0.514		
Diabetes	0.78 (0.22–2.80)	0.705		
Chronic kidney disease	0.62 (0.16–2.44)	0.491		
Body weight change^a^	1.06 (0.89–1.26)	0.503		
LVEF	1.01 (0.97–1.05)	0.663		
NT-pro-BNP^b^	1.01 (0.59–1.71)	0.981		
Total re-intubation during the hospital stay				
Positive B-line	3.88 (1.50–10.05)	0.005	5.54 (1.60–19.18)	0.007
Total number of B-line	1.06 (1.02–1.11)	0.004	1.11 (1.02–1.21)	0.018
Age	1.02 (0.98–1.06)	0.310		
Hypertension	0.94 (0.38–2.33)	0.894		
Diabetes	0.99 (0.41–2.42)	0.990		
Chronic kidney disease	1.27 (0.52–3.09)	0.604		
Body weight change^a^	1.02 (0.91–1.14)	0.777		
LVEF	1.01 (0.99–1.04)	0.354		
NT-pro-BNP^b^	1.26 (0.82–1.94)	0.295		

Variables of positive B-line and the total number of B-line were analyzed independently by adjusting the following variables: age, hypertension, diabetes, chronic kidney disease, body weight change, LVEF, and NT-pro-BNP.

CI, confidential interval; LVEF, left ventricular ejection fraction; NT-pro-BNP, N-terminal pro-B-type natriuretic peptide; OR, odds ratio.

^a^ Body weight change was calculated as the change between the value at the time of intubation and extubation.

^b^ NT-pro-BNP level was analyzed with log-transformed value.

**Table 4:** Sensitivity, specificity, positive and negative predictive values of a positive LUS result.

Endpoint	Sensitivity	Specificity	PPV	NPV
Re-intubation within 72 h	81.8%	59.3%	14.1%	97.6%
Total re-intubation during the hospital stay	70.8%	61.5%	26.6%	91.5%

LUS, lung ultrasound; NPV, negative predictive value; PPV, positive predictive value.

## Discussion

This study is the first to investigate the predictive value of B-lines detected by LUS in patients receiving invasive MV due to acute decompensated HF. Our findings suggest that a positive LUS result is associated with a higher risk of re-intubation during both the early period after extubation and the entire period of hospital stay, as well as longer ICU and hospital stays. Moreover, the risk of re-intubation increased with an increase in a total B-line count. A B-line count was correlated with the NT-pro-BNP level; however, a positive LUS result, but not the total count of B-lines or NT-pro-BNP, was independently associated with re-intubation risk after weaning. Given the high NPV of a positive LUS result, it may serve as a useful parameter to exclude the possibility of re-intubation before deciding on extubation for HF patients on invasive MV.

A subset of patients with HF may develop respiratory failure that requires invasive MV due to either decompensation of pre-existing HF or newly diagnosed HF. HF itself is a risk factor for weaning failure (16), and positive fluid balance, high levels of NPs, and echocardiographic parameters of diastolic function have been shown to predict weaning failure in patients with HF (17–19). Moreover, even though many of these patients can be successfully weaned off with appropriate therapy, a significant proportion may experience re-intubation (3, 4), which is strongly associated with adverse in-hospital outcomes (20). Adequate decongestion is important to avoid re-intubation in the HF population, as pulmonary edema reduces lung compliance and can act as a major driver of respiratory failure (21, 22). In contrast, insufficient decongestion, reflected by less body weight reduction, has been associated with a higher risk of re-intubation (4). However, it is important to note that conventional parameters such as body weight change, echocardiography, and NPs are indirect indicators of pulmonary decongestion.

LUS is a quick and straightforward method that can be completed within a few minutes. A B-line, as measured by LUS, is a hyperechoic artifact that extends vertically from the pleural line and represents a sonographic sign of pulmonary edema with high sensitivity and specificity (23, 24). It has been well correlated with PCWP and interstitial edema on computed tomography (25, 26), and its use has been reported in the diagnosis of suspected HF, the assessment of adequacy in decongestive therapy, and the prediction of outcomes of chronic HF (12, 27–30). Although the clinical value of B-lines has been previously reported for HF patients in pre-hospital or ambulatory settings, its usefulness in patients with decompensated HF dependent on MV has not been studied extensively. Previous studies reported conflicting results for LUS to predict outcomes after extubation, while loss of lung aeration may predict post-extubation distress (11, 31). However, these studies primarily included patients with non-cardiac causes for the initiation of MV. In the present study, we demonstrated that the B-lines detected by LUS were an independent predictor of re-intubation after MV weaning in HF patients. Although the number of early re-intubations in this study was small (a total of 11), a higher count of total B-lines indicated an increased risk of re-intubation, supporting the predictive benefit of B-lines by LUS in the current study. Furthermore, the high negative predictive value of B-lines suggests that HF patients with negative LUS results who are scheduled for extubation can be safely weaned off without a significant risk of re-intubation. However, given the relatively low positive predictive value of B-lines for re-intubation risk, they cannot reliably predict the actual risk of re-intubation. Therefore, the current study results do not provide information for identifying patients at a higher risk of re-intubation.

NPs increase in response to volume overload and are recognized as predictors of outcomes in HF patients. Furthermore, elevated levels of NPs are predictive of weaning failure in patients on MV (32). However, the correlation between NPs and PCWP has been reported to be weak in patients admitted to the ICU (33, 34). Forfia et al. (33) reported NP level cannot reliably predict left ventricular filling pressure in the setting of critical care, especially under circumstance of impaired renal function. In addition, NPs can be elevated in various conditions, including worsening renal function, which is frequently present during episodes of decompensated HF (33, 35). In our study, a significant yet weak correlation was observed between B-line status and NT-pro-BNP, consistent with previous studies. However, traditional diagnostic tools, such as chest radiography and NPs often have limited value in ICU settings. Notably, sonographic findings indicative of interstitial edema correlate with PCWP, even under mechanical ventilation (26, 36). This is particularly relevant considering our study's finding that a positive LUS exam can predict early re-intubation. Therefore, B-line measurements may provide valuable information to guide safe weaning decisions.

Our study had several limitations. First, weaning failure and subsequent re-intubation is a complex outcome that is influenced by various factors, including cardiac condition, interstitial edema, and neuromuscular, metabolic, psychological, and nutritional factors (13). Therefore, the B-line result alone cannot be the sole determinant in deciding whether to extubate. Nevertheless, adequate decongestion is essential for HF patients before being weaned off MV, and this can be easily detected through LUS. Second, there are numerous methods used to obtain LUS images from various regions. We chose to scan eight regions, including the lateral side of the thorax. The sonographic distribution of B-lines in cardiogenic pulmonary edema is frequently not even, possibly being accentuated in the basal part of the lung, which can be detected by examining the lateral side of the thorax in a supine position (37). Third, the correlation between a total B-line count and the NT-pro-BNP level was weak, and its significance was higher in the study population not dependent on dialysis than in dialysis-dependent patients. However, NT-pro-BNP levels can be falsely elevated in dialysis-dependent patients, which limits its use in making weaning decisions in these populations (35). Fourth, although multiple B-lines suggest pulmonary interstitial edema, they can also be present in numerous conditions, including acute lung injury, pulmonary fibrosis, and even in normal lung in small numbers. Therefore, sole guidance by LUS for MV weaning cannot be warranted. Fifth, the study population was limited to individuals with HF. Consequently, our study findings cannot be extrapolated to encompass all ICU patients with MV. Last, despite its high NPV, the low positive predictive value may limit the clinical utility for identifying patients at high risk of re-intubation who require further decongestive therapy. Furthermore, the low rate of the primary outcome may affect its high NPV, which should be replicated in other studies.

## Conclusions

This study demonstrated that B-lines, measured by LUS before extubation in patients receiving invasive MV due to acute decompensated HF, can predict the risk of re-intubation. Also, a negative LUS result was found to be highly predictive of safe weaning from MV without significant risk of re-intubation. Therefore, B-line measurement may assist in guiding decisions on MV weaning in this population.

## Data Availability

The raw data supporting the conclusions of this article will be made available by the authors, without undue reservation.
