# Supplementary material for: B-lines by lung ultrasound as a predictor of re-intubation in mechanically ventilated patients with heart failure
Source: Front Cardiovasc Med. 2024 Feb 8;11:1351431. doi: 10.3389/fcvm.2024.1351431 (PMC10881858; doi:10.3389/fcvm.2024.1351431)
Supplement: Supplementary file 1 [file Datasheet1.docx]

**SUPPLEMENTARY MATERIALS**

Hyun J, Kim A, Lee SE, Kim MS. B-line by Lung Ultrasound as a Predictor of Re-intubation in Mechanically Ventilated Patients with Heart Failure

**Supplementary Figures**

**Figure 1.** Distribution of a total count of B-line measurements**Figure 2.** Correlation Between a B-line count and NT-pro-BNP level

**Supplementary Tables**

**Table 1.** B-line count and proportion of positive results at each region of the thorax

**Table 2.** B-line count and proportion of positive results in each region according to the B-lines result

**Table 3.** Rates of re-intubation according to the tertile of B-line count

**Supplementary Figure 1**. Distribution of a total count of B-line measurements


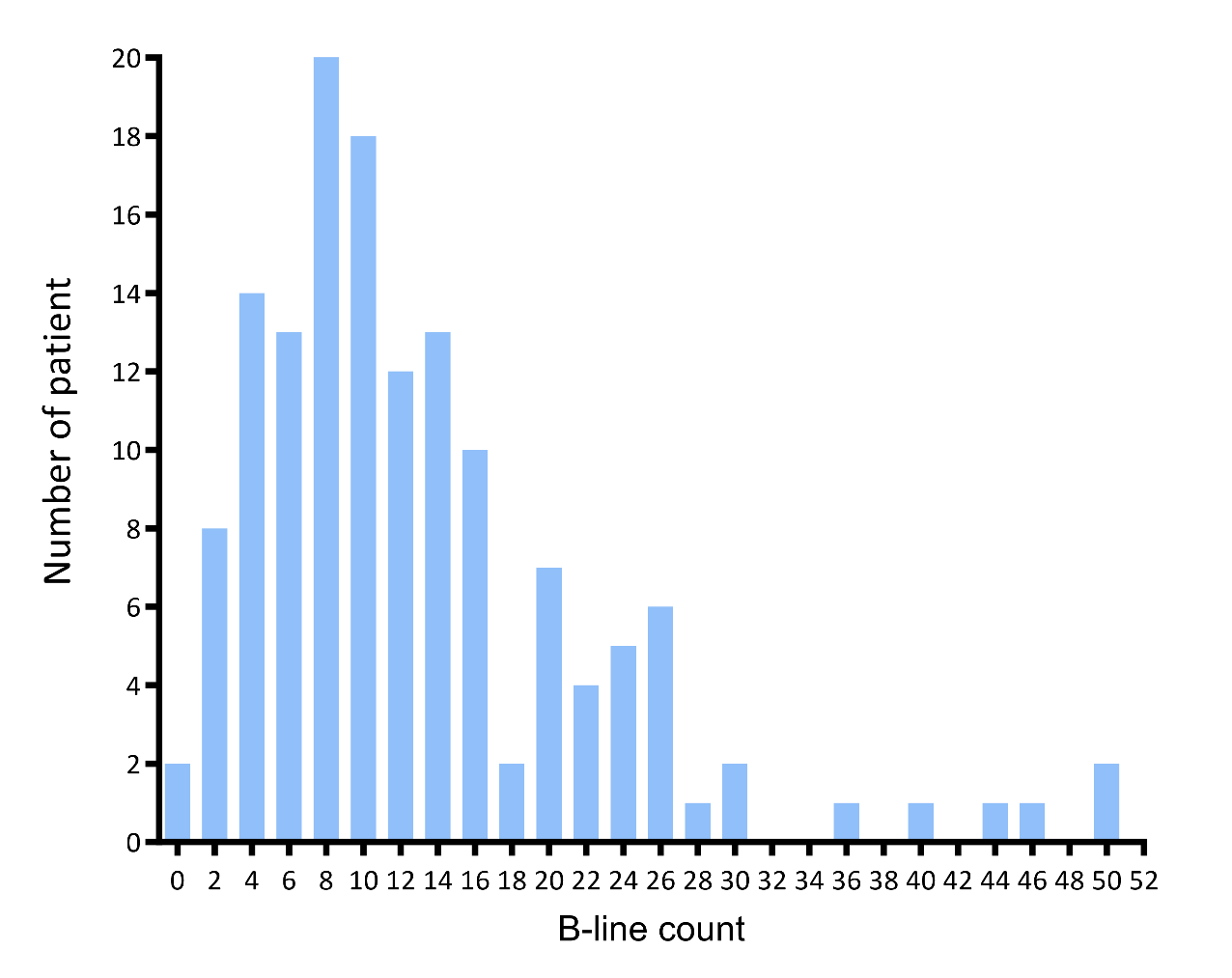


**Supplementary Figure 2. Correlation Between a B-line count and NT-pro-BNP level**


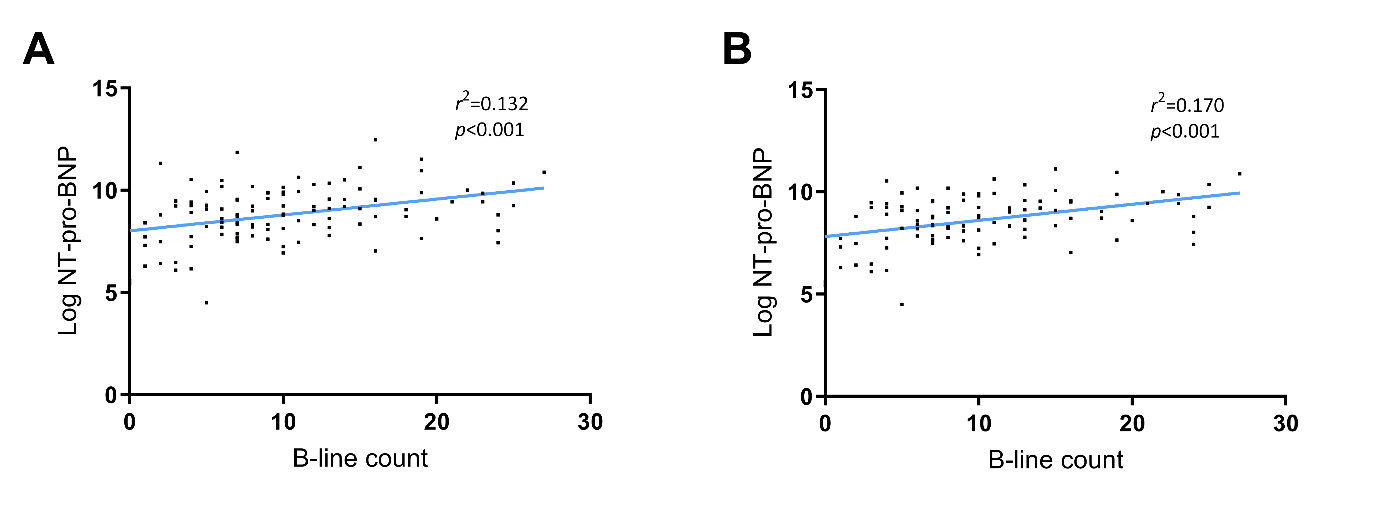


(A) Whole study population. (B) Patients not dependent on dialysis.

*NT-pro-BNP level was transformed into its log value due to a skewed distribution.

NT-pro-BNP = N-terminal pro-B-type natriuretic peptide.

**Supplementary Table 1**. B-line count and proportion of positive results at each region of the thorax

| **Region** | **Number of B-line,**  **Median (IQR)** | **Proportion of positive region*****,**  **n (%)** |
| --- | --- | --- |
| Total | 10 (6–16) | 292/1168 (25.0) |
| Right upper medial | 1 (0–2) | 26 (17.8) |
| Right lower medial | 1 (0–2) | 32 (21.9) |
| Right upper lateral | 1 (1–2) | 33 (22.6) |
| Right lower lateral | 2 (1–3) | 64 (43.8) |
| Left upper medial | 0 (0–2) | 19 (13.1) |
| Left lower medial | 0 (0–1) | 14 (9.6) |
| Left upper lateral | 1 (0–3) | 37 (25.3) |
| Left lower lateral | 2 (1–3) | 67 (45.9) |

*Positive region was defined as the region that had 3 or more B-lines.

IQR = interquartile range.

**Supplementary Table 2**. B-line count and proportion of positive results in each region according to the B-line result

| **Region** | **Positive B-line group**  **N=64** | **Negative B-line group**  **N=82** |
| --- | --- | --- |
| **Number of B-line, median (IQR)** | | |
| Right upper medial | 2 (0–3) | 0 (0–1) |
| Right lower medial | 2 (1–4) | 1 (0–2) |
| Right upper lateral | 2 (1–3) | 1 (0–1) |
| Right lower lateral | 3 (3–4) | 1 (1–2) |
| Left upper medial | 2 (0–3) | 0 (0–1) |
| Left lower medial | 1 (0–2) | 0 (0–1) |
| Left upper lateral | 3 (1–4) | 1 (0–1) |
| Left lower lateral | 3 (3–4) | 2 (1–2) |
| **Proportion of positive region, n (%)** | | |
| Right upper medial | 21 (32.8) | 5 (6.1) |
| Right lower medial | 28 (43.8) | 4 (4.9) |
| Right upper lateral | 31 (48.4) | 2 (2.4) |
| Right lower lateral | 53 (82.8) | 11 (13.4) |
| Left upper medial | 19 (29.7) | 0 (0.0) |
| Left lower medial | 13 (20.3) | 1 (1.2) |
| Left upper lateral | 33 (51.6) | 4 (4.9) |
| Left lower lateral | 54 (84.4) | 13 (15.9) |

IQR = interquartile range.

**Supplementary Table 3.** Rates of re-intubation according to the tertile of B-line count

|  | **1st tertile**  **N=50** | **2nd tertile**  **N=48** | **3rd tertile**  **N=48** | **p Value** |
| --- | --- | --- | --- | --- |
| Number of B-line | 0–7 | 8–13 | ≥14 |  |
| Re-intubation within 72 hours | 1 (2.0) | 3 (6.3) | 7 (14.6) | 0.055 |
| Tertile 1st vs. 2nd |  |  |  | 0.357 |
| Tertile 2nd vs. 3rd |  |  |  | 0.181 |
| Tertile 1st vs. 3rd |  |  |  | 0.029 |
| Total re-intubation during admission | 2 (4.0) | 6 (12.5) | 16 (33.3) | <0.001 |
| Tertile 1st vs. 2nd |  |  |  | 0.155 |
| Tertile 2nd vs. 3rd |  |  |  | 0.015 |
| Tertile 1st vs. 3rd |  |  |  | <0.001 |
